# Palmitate potentiates the SMAD3-PAI-1 pathway by reducing nuclear GDF15 levels

**DOI:** 10.1007/s00018-024-05571-y

**Published:** 2025-01-18

**Authors:** Marta Montori-Grau, Emma Barroso, Javier Jurado-Aguilar, Mona Peyman, Walter Wahli, Xavier Palomer, Manuel Vázquez-Carrera

**Affiliations:** 1 Department of Pharmacology, Toxicology and Therapeutic Chemistry, Faculty of Pharmacy and Food Sciences, Unitat de Farmacologia, Universitat de Barcelona, Av. Joan XXIII 27-31, 08028 Barcelona, Spain; 2 Institute of Biomedicine, University of Barcelona (IBUB), 08028 Barcelona, Spain; 3 Spanish Biomedical Research Center in Diabetes and Associated Metabolic Diseases (CIBERDEM), Instituto de Salud Carlos III, 28029 Madrid, Spain; 4 Pediatric Research Institute-Hospital Sant Joan de Déu, 08950 Esplugues de Llobregat, Spain; 5 Center for Integrative Genomics, University of Lausanne, 1015 Lausanne, Switzerland; 6 Lee Kong Chian School of Medicine, Nanyang Technological University, Singapore, 308232 Singapore; 7 ToxAlim (Research Center in Food Toxicology), INRAE, UMR1331, 31300 Toulouse Cedex, France

**Keywords:** GDF15, Insulin resistance, SMAD3, PAI-1, Palmitate, Oleate, Muscle

## Abstract

Nuclear growth differentiation factor 15 (GDF15) reduces the binding of the mothers’ against decapentaplegic homolog (SMAD) complex to its DNA-binding elements. However, the stimuli that control this process are unknown. Here, we examined whether saturated fatty acids (FA), particularly palmitate, regulate nuclear GDF15 levels and the activation of the SMAD3 pathway in human skeletal myotubes and mouse skeletal muscle, where most insulin-stimulated glucose use occurs in the whole organism. Human LHCN-M2 myotubes and skeletal muscle from wild-type and *Gdf15*^*−/−*^ mice fed a standard (STD) or a high-fat (HFD) diet were subjected to a series of studies to investigate the involvement of lipids in nuclear GDF15 levels and the activation of the SMAD3 pathway. The saturated FA palmitate, but not the monounsaturated FA oleate, increased the expression of *GDF15* in human myotubes and, unexpectedly, decreased its nuclear levels. This reduction was prevented by the nuclear export inhibitor leptomycin B. The decrease in nuclear GDF15 levels caused by palmitate was accompanied by increases in SMAD3 protein levels and in the expression of its target gene *SERPINE1*, which encodes plasminogen activator inhibitor 1 (PAI-1). HFD-fed *Gdf15*^−/−^ mice displayed aggravated glucose intolerance compared to HFD-fed WT mice, with increased levels of SMAD3 and PAI-1 in the skeletal muscle. The increased PAI-1 levels in the skeletal muscle of HFD-fed *Gdf15*^−/−^ mice were accompanied by a reduction in one of its targets, hepatocyte growth factor (HGF)α, a cytokine involved in glucose metabolism. Interestingly, PAI-1 acts as a ligand of signal transducer and activator of transcription 3 (STAT3) and the phosphorylation of this transcription factor was exacerbated in HFD-fed *Gdf15*^−/−^ mice compared to HFD-fed WT mice. At the same time, the protein levels of insulin receptor substrate 1 (IRS-1) were reduced. These findings uncover a potential novel mechanism through which palmitate induces the SMAD3-PAI-1 pathway to promote insulin resistance in skeletal muscle by reducing nuclear GDF15 levels.

**Supplementary Information:**

The online version contains supplementary material available at 10.1007/s00018-024-05571-y.

## Introduction

Growth differentiation factor 15 (GDF15) is a divergent member of the transforming growth factor-β (TGF-β) superfamily, acting as a stress-induced cytokine [1]. In fact, under conditions of cellular stress, such as in several diseases (especially in cancer, cardiovascular disease, obesity, diabetes, mitochondrial diseases, and aging), serum GDF15 levels are remarkably increased [2–4]. Most of the effects of this cytokine are mediated by its binding to glial cell line-derived neurotrophic factor (GDNF)-family receptor α-like (GFRAL) and its coreceptor RET. This receptor is solely expressed in the neurons of the area postrema and solitary tract nucleus, which are brain regions involved in appetite and weight regulation [5–8]. The activation of the GDF15-GFRAL pathway reduces food intake and body weight through nausea, emesis, and taste aversion [9, 10]. In addition, GDF15 causes peripheral metabolic effects, independent of GFRAL, through yet-to-be discovered mechanisms [11–14].

GDF15 may be present in different forms within the cell. It is synthesized in the cytoplasm as a biologically inactive precursor called pre-pro-GDF15. Pro-GDF15 is generated after cleavage of the N-terminal signal peptide. Eventually, pro-GDF-15 is cleaved into the mature GDF15 to be secreted by the cells. Pro-GDF15 translocates to the nucleus under the influence of a domain comprising both a nuclear localization signal and a nuclear export signal [2, 15, 16]. Once in the nucleus, pro-GDF15 interacts with the mothers against decapentaplegic homolog (SMAD) complex, reducing its binding to its DNA target elements. This attenuates the TGF-β1-induced signaling pathway, thereby lowering the expression of the genes upregulated by this growth factor and causing alterations in cell migration and invasion [2, 15]. These findings suggest that nuclear GDF15 acts as an inhibitor of the TGF-β1 pathway. However, it remains unknown which stimuli regulate GDF15 nuclear export and whether the changes in nuclear GDF15 levels affect metabolism.

*GDF15* expression is upregulated by different transcription factors, including p53, early growth response protein 1 (EGR1), nuclear factor erythroid 2–related factor 2, activating transcription factor 4 (ATF4), and C/EBP homologous protein (CHOP) [3, 13, 17]. CHOP plays a key role in upregulating *GDF15* expression via the integrated stress response (ISR) [3]. This is a signaling pathway activated by diverse stress stimuli, including endoplasmic reticulum (ER) stress and the subsequent adaptive unfolded protein response (UPR). This adaptive response attempts to restore ER homeostasis or, if the stress becomes chronic, induces cell death [18]. It is activated through the phosphorylation of eukaryotic initiation factor-2α, which increases ATF4 activity, ultimately leading to increased CHOP levels. Interestingly, saturated fatty acids (FAs) such as palmitate, the primary saturated FA in most diets, activate the ER stress-UPR pathway, increasing the expression levels of GDF15 via CHOP and promoting insulin resistance and type 2 diabetes mellitus (T2DM) [19–21]. By contrast, the most common monounsaturated FA in daily nutrition, oleate, does not cause ER stress [22, 23] and increases insulin sensitivity [19]. The mechanisms underlying the differences between saturated and monounsaturated FAs in their effects on insulin resistance have not been fully elucidated. In this study, we examined how saturated and monounsaturated FAs may affect the nuclear levels of GDF15, the activation of the SMAD pathway, and the development of insulin resistance in human LHCN-M2 myotubes and mouse skeletal muscle, a tissue where most of the insulin-stimulated glucose use occurs [24, 25]. Our findings show that the saturated FA palmitate and the monounsaturated FA oleate differ in how they affect the levels of CHOP and GDF15 in LHCN-M2 myotubes. Interestingly, palmitate causes a sustained reduction in the nuclear levels of GDF15 in LHCN-M2 myotubes, which is prevented by the nuclear export inhibitor leptomycin B. Oleate has no effect on the GDF15 export process. In addition, exposure of the cells to palmitate increases SMAD3 levels and causes a robust elevation in the expression of its target gene *SERPINE1* and its encoded protein plasminogen activator inhibitor 1 (PAI-1), with this effect prevented by leptomycin B. Consistent with this, *Gdf15*^−/−^ mice fed a high-fat diet (HFD) show increased glucose intolerance and elevated levels of SMAD3 and PAI-1 in their skeletal muscle compared to HFD-fed wild-type (WT) mice. Overall, these findings suggest that the saturated FA palmitate modulates the SMAD3-PAI-1 pathway by regulating GDF15 nuclear levels. By contrast, oleate does not, thereby unveiling a new important difference between saturated and monounsaturated FAs that may contribute to their unequal capacity to induce insulin resistance.

## Materials and methods

### Mice and in vivo studies

Male *Gdf15*^−/−^ and WT mice (10–12 weeks old, C57BL/6/129/SvJ background), obtained from the Johns Hopkins University School of Medicine (USA), were randomly distributed into four experimental groups: standard diet (STD)-WT; Western-type HFD (60% kcal from fat, product D12492, Research Diets Inc.)-WT; STD-*Gdf15*^−/−^, and HFD-*Gdf15*^−/−^. At the end of the 16-week treatment, the mice were sacrificed, and skeletal muscle (gastrocnemius) samples were obtained and frozen in liquid nitrogen before storage at −80 °C.

For the glucose tolerance test (GTT), animals fasted for 6 h received 2 g/kg of body weight of glucose via an intraperitoneal injection and blood was collected from the tail vein after 0, 15, 30, 60, and 120 min. Animal experimentation complied with the Guide for the Care and Use of Laboratory Animals published by the US National Institutes of Health (8th Edition: National Academies Press; 2011). All procedures were approved by the Bioethics Committee of the University of Barcelona, as stated in Law 5/21 July 1995 passed by the Generalitat de Catalunya. The animals were treated humanely and all efforts were made to minimize both animal numbers and suffering.

### Cell culture

The human LHCN-M2 myoblast cell line, which is used to investigate skeletal muscle development and metabolism, was kindly provided by Dr. W.E. Wright and was cultured and differentiated for 5 days into myotubes, as previously described [26, 27]. Lipid-containing media were prepared by conjugating palmitic acid (P0500, Sigma-Aldrich, Madrid, Spain) or oleic acid (O1008, Sigma-Aldrich) with fatty acid-free bovine serum albumin (A3803, Sigma-Aldrich), as previously described [23]. Control cells were incubated with fatty acid-free bovine serum albumin. Cells were co-incubated with the FA and 10 nM leptomycin B (L2913, Sigma-Aldrich) or 10 µM SIS3 (ref. 566,405, Calbiochem, San Diego, CA, USA) for the indicated times. To evaluate the insulin signaling pathway, LHCN‑M2 myotubes were incubated for 1 h with 5 nM leptomycin B or 10 µM sulindac sulfide (574,102, Sigma-Aldrich) and then incubated in the presence or in the absence of 0.5 mM palmitate for additional 16 h. When indicated, cells were stimulated with or without 100 nM of insulin (Ins) for the last 10 min.

### RNA extraction, reverse transcription (RT) and real-time PCR

Total RNA from cultured cells was extracted with the Quick-RNA Miniprep Kit (Zymo Research, Irvine, CA, USA), while total RNA from mouse muscle was extracted with the RNeasy Mini Kit (Qiagen Venlo, the Netherlands), following the manufacturer’s instructions. 0.5 µg of total RNA was retrotranscribed with TaqMan RT reagents from Applied Biosystems (Foster City, CA, USA) using random hexamers. Real-time PCR was performed in the LightCycler 480 SW 1.5 instrument, using the LightCycler 480 Probes Master and probes for the selected human or mouse genes from Applied Biosystems. The GAPDH gene was used as the endogenous control to normalize the crossing point (Cp) for each probe assay. The relative gene expression was calculated as 2^−ΔCp^ and the gene fold change was calculated with the 2^−ΔΔCp^ method.

### Immunoblotting

LHCN-M2 cell total protein extracts were prepared by scraping the cell monolayers from 6-cm dishes or 6-well plates into 100 or 75 µl of homogenization buffer, respectively, containing RIPA buffer (Sigma-Aldrich), 50 mM NaF, 1 mM phenylmethylsulfonyl fluoride (PMSF), 10 mM Na_3_VO_4_, 10 mM nicotinamide, and a protease inhibitor cocktail (Sigma-Aldrich). For in vivo studies, 50 mg of mouse muscle were homogenized with a polytron in 500 µl of homogenization buffer. Lysates were then gently rocked for 60 min at 4 °C and centrifuged at 10,000*g* for 30 min at 4 °C.

Cytoplasmic and nuclear fractions were obtained by scraping the cell monolayers from 10-cm dishes or 50 mg of mouse muscle into 500 µl of homogenization buffer containing 5 mM MgCl_2_, 50 mM Tris–HCl (pH 4.7), 250 mM sucrose and a protease inhibitor cocktail (Sigma Aldrich), as previously described [28].

An aliquot of the supernatant was used to measure protein concentrations with the Pierce BCA Protein Assay Kit (Pierce, Thermo Fisher Scientific, Waltham, MA, USA). Proteins were resolved by SDS/-PAGE (10%, w/v). Immunoblotting was performed with antibodies against α-actinin (sc-15335; Santa Cruz Biotechnology Inc., Dallas, TX, USA), total (#9272) and phospho-Akt (Ser^473^) (#587F11) (Cell Signaling Technology, Danvers, MA, USA), α-tubulin (sc-8035; Santa Cruz Biotechnology Inc.), CHOP (#2895, Cell Signaling Technology), GAPDH (sc-365062, Santa Cruz Biotechnology Inc.), GDF15 (sc-515675; Santa Cruz Biotechnology Inc.), hepatocyte growth factor (HGF)α (sc-374422; Santa Cruz Biotechnology Inc.), histone H2B (sc-525808; Santa Cruz Biotechnology Inc.), insulin receptor substrate 1 (IRS-1) (sc-7200; Santa Cruz Biotechnology Inc.), lamin B1 (#12586, Cell Signaling Technology), PAI-1 (#11907, Cell Signaling Technology), total (#9513, Cell Signaling Technology) and phospho-SMAD3 (Ser^423^/^425^) (#9520, Cell Signaling Technology) and total (#4904, Cell Signaling Technology) and phospho-STAT3 (Tyr^705^) (#9145, Cell Signaling Technology).

### Cytolocation of the fusion construct GFP-GDF15

To cytolocate the green fluorescent protein (GFP)-GDF15 construct within the cell nuclei, we used 90–95% confluent LHCN-M2 myotubes grown on coverslips and transfected with 3 µg of pGFP-GDF15 [human GDF15 Expression-Ready ORF clone (GFP, C-terminus), Cat.ID: LS-N19467 from LifeSpan BioScience Inc., Shirley, MA, USA] with the aid of Lipofectamine™ 2000 Transfection Reagent (Thermo Fisher Scientific) during 24 h following manufacturer’s instructions. pEGFP-C2 (BD Biosciences, San Jose, USA) was used to express GFP. Cells were incubated in the presence or absence (control) of 0.5 mM palmitate (Pal) with or without 10 nM leptomycin B or 0.5 mM oleate for 16 h. Then, cells were fixed with 4% paraformaldehyde for 15 min at room temperature and the nuclei were stained with Hoechst (1 µg/ml). Cell preparations were mounted with ProLong Gold (Invitrogen, Paisley, UK) and analyzed with a A Zeiss LSM880 laser scanning spectral confocal microscope (Carl Zeiss, Jena, Germany).

### Statistics

Data are presented as the mean ± SEM. Significant differences were assessed by Student’s t-test or one-way and two-way ANOVA, according to the number of groups being compared, using the GraphPad Prism program (V8.4.3) (GraphPad Software Inc., San Diego, CA, USA). The Tukey–Kramer multiple comparison post-hoc test was performed when significant differences were found by ANOVA. Differences were considered significant at *P* < 0.05. The results for gene expression (fold change from the real-time PCR analysis) were examined with the Relative Expression Software Tool (REST).

## Results

### Palmitate, but not oleate, increases GDF15 expression and CHOP protein levels in human LHCN-M2 myotubes

**Fig. 1:**
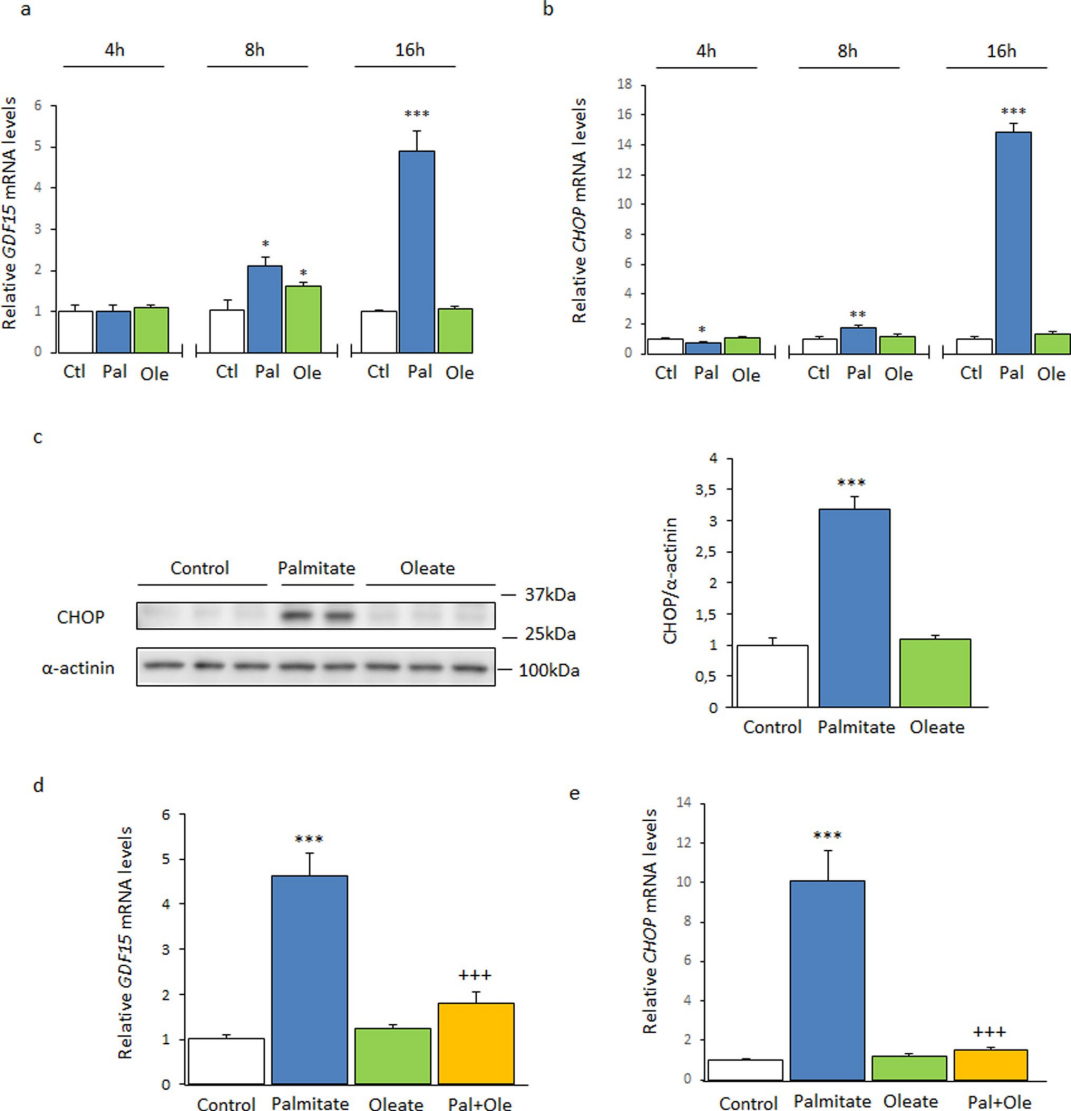
Palmitate, but not oleate, increases the expression of *GDF15* and *CHOP* in human LHCN-M2 myotubes. Time course of **a** *GDF15* and **b** *CHOP* mRNA levels in human LHCN-M2 myotubes incubated in the presence or absence (control) of 0.5 mM palmitate (Pal) or 0.5 mM oleate (Ole) (n = 3–10). **c** Immunoblot analysis of CHOP in human LHCN-M2 myotubes incubated in the presence or absence (control) of 0.5 mM palmitate (Pal) or 0.5 mM oleate (Ole) for 16 h (n = 5). **d** *GDF15* and **e** *CHOP* mRNA levels in human LHCN-M2 myotubes incubated for 16 h in the absence (control) or presence of different fatty acids: 0.5 mM palmitate, 0.5 mM oleate or 0.5 mM palmitate supplemented with 0.3 mM oleate (n = 5). Data are presented as the mean ± SEM. *p < 0.05, **p < 0.01, and ***p < 0.001 *versus* control. ^+++^*p* < 0.001 versus palmitate-treated cells

First, we examined the effects of the saturated FA palmitate and the monounsaturated FA oleate on the expression of *GDF15* in human LHCN-M2 myotubes. A time-course study showed that palmitate caused a robust increase in the mRNA levels of *GDF15* after 16 h of exposure, whereas oleate only transiently increased GDF15 after 8 h (Fig. 1a). Likewise, incubation of the cells with palmitate increased the expression and protein levels of CHOP, but oleate did not affect the levels of this ER stress marker (Fig. 1b, c). Since we have previously reported that co-incubation of myotubes with a lower concentration of oleate attenuates the effects of palmitate on ER stress [23], we assessed whether 0.3 mM oleate attenuated the increase in *GDF15* expression caused by 0.5 mM palmitate. Interestingly, cells co-incubated with oleate and palmitate showed a lower expression of *GDF15* than those incubated with only palmitate (Fig. 1d). The same trend was observed for CHOP mRNA levels (Fig. 1e). These findings suggest that palmitate induces ER stress and the subsequent increase in CHOP levels might ultimately upregulate the levels of the stress cytokine GDF15. By contrast, oleate does not produce these changes and even has the capacity to attenuate the ER stress caused by palmitate and, thus, decrease the expression of *GDF15*.

### Palmitate, but not oleate, causes a sustained reduction in nuclear GDF15 levels in human LHCN-M2 myotubes

**Fig. 2:**
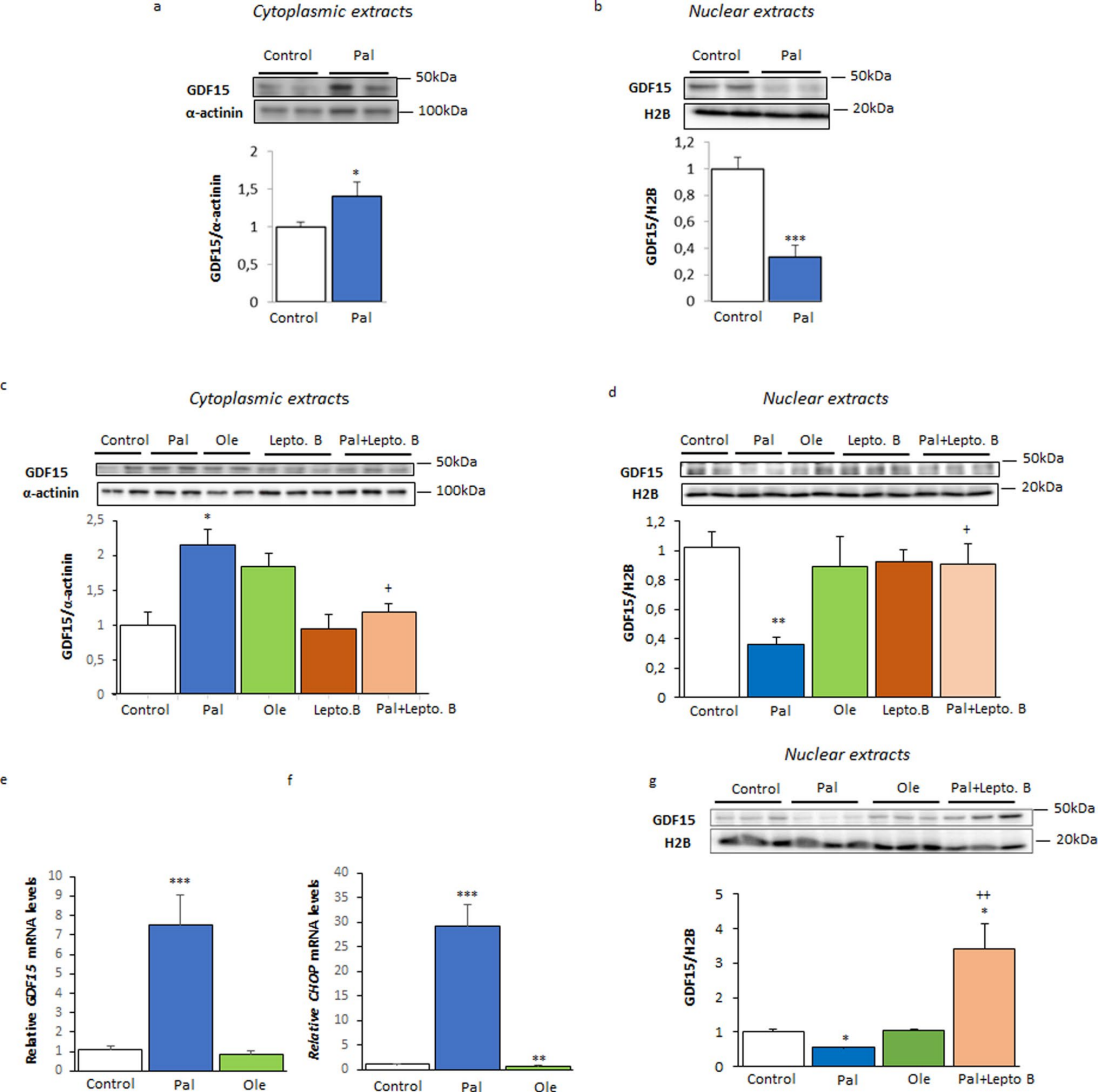
Palmitate, but not oleate, reduces the nuclear protein levels of GDF15 in human LHCN-M2 myotubes. Immunoblot analysis of GDF15 in **a** cytoplasmic and **b** nuclear extracts from human LHCN-M2 myotubes incubated in the presence or absence (control) of 0.5 mM palmitate (Pal) for 16 h (n = 6). Immunoblot analysis of GDF15 in **c** cytoplasmic and **d** nuclear extracts from human LHCN-M2 myotubes incubated in the presence or absence (control) of 0.5 mM palmitate (Pal) with or without 10 nM leptomycin B (Lepto B) or 0.5 mM oleate (Ole) for 16 h (n = 4). **e** *GDF15* and **f** *CHOP* mRNA levels in human LHCN-M2 myotubes incubated for 48 h in the presence or absence (control) of 0.5 mM palmitate (Pal) or 0.5 mM oleate (Ole) (n = 6). **g** Immunoblot analysis of GDF15 in nuclear extracts from human LHCN-M2 myotubes incubated in the presence or absence (control) of 0.5 mM palmitate (Pal) with or without 10 nM leptomycin B or 0.5 mM oleate (Ole) for 48 h (n = 3). Data are presented as the mean ± SEM. *p < 0.05, **p < 0.01, and ***p < 0.001 *versus* control. ^+^*p* < 0.05, ^++^*p* < 0.01, and ^+++^*p* < 0.001 *vs.* palmitate-treated cells

Next, we evaluated the effects of palmitate and oleate on cytoplasmic and nuclear levels of GDF15 in LHCN-M2 myotubes. Since it has been reported that only pro-GDF15 is present inside cells (with no mature form detected) [15], the cytoplasmic and nuclear GDF15 mentioned in this study corresponds to pro-GDF15. α-Tubulin and H2B were used as controls for the cytoplasmic and nuclear fractions, respectively (Supplementary Fig. 1a). In agreement with the gene expression findings, cells exposed to palmitate for 16 h showed increased protein levels of cytoplasmic GDF15 (Fig. 2a). By contrast, palmitate caused a marked reduction in the nuclear levels of GDF15 (Fig. 2b). Of note, the increase in cytoplasmic GDF15 levels (Fig. 2c) and decrease in nuclear GDF15 levels (Fig. 2d) caused by palmitate were not observed in the cells incubated with oleate. The effect of palmitate was prevented by the specific and potent nuclear export inhibitor leptomycin B. This compound alkylates and inhibits chromosome regional maintenance 1 (CRM1, also known as exportin 1), which is required for nuclear export. In agreement with the findings obtained after 16 h of incubation, cells exposed to palmitate for 48 h showed increased *GDF15* (Fig. 2e) and *CHOP* (Fig. 2f) expression. By contrast, oleate did not have any effect. Remarkably, incubation of the cells with palmitate for 48 h caused a persistent reduction in nuclear GDF15 levels that was again prevented by leptomycin B (Fig. 2g). Of note, oleate did not reduce nuclear GDF15 levels (Fig. 2g).

**Fig. 3:**
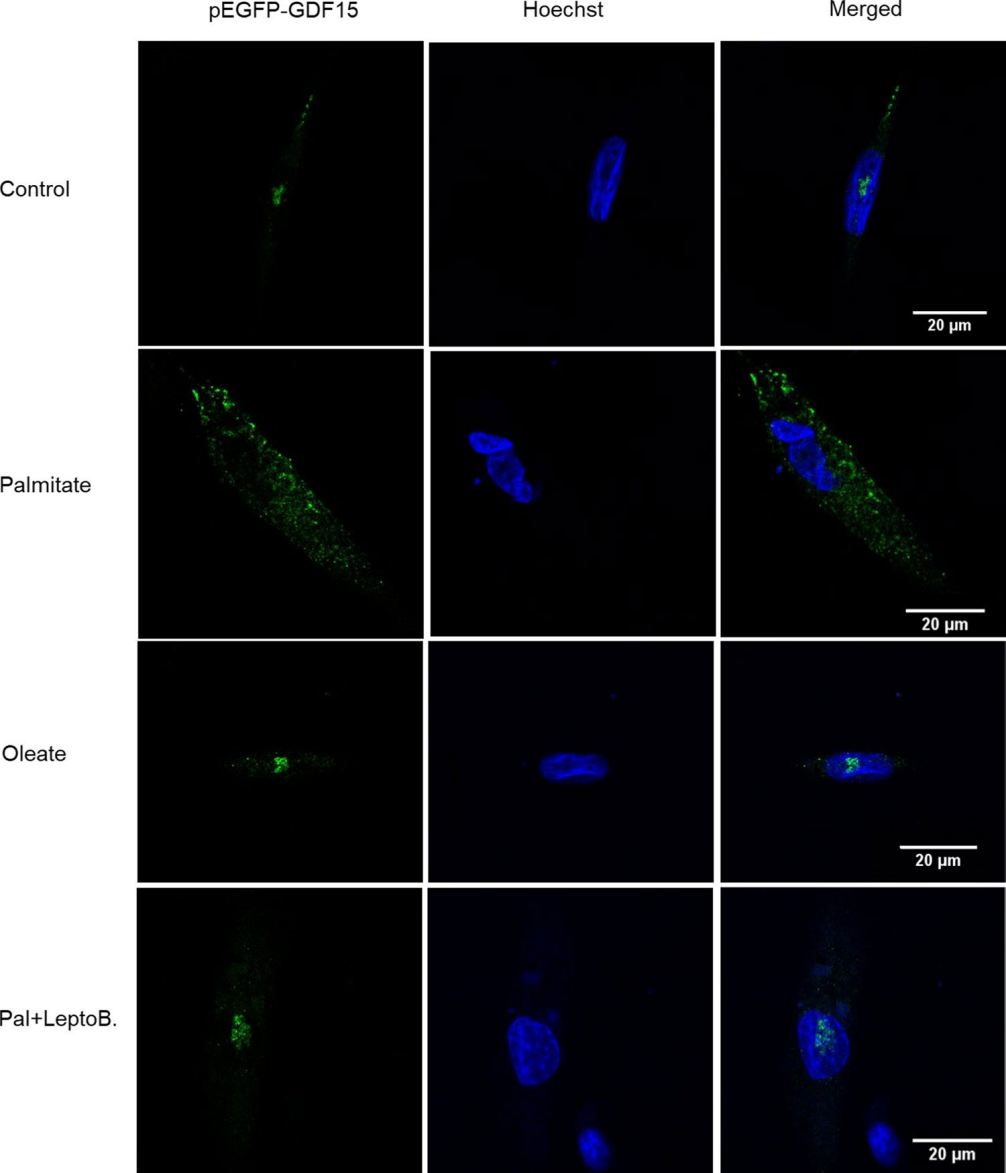
Cytolocalization of GFP-GDF15 in LHCN-M2 myotubes. LHCN-M2 cells were transfected with plasmids pGFP (not shown) or pGFP-GDF15 and incubated in the presence or absence (control) of 0.5 mM palmitate (Pal) with or without 10 nM leptomycin B (LeptoB.) or 0.5 mM oleate for 16 h. The representative image shows the fluorescent signal of GFP and blue fluorescence of Hoechst and the co-localization of the signal of GFP and Hoechst

To corroborate that palmitate reduces GDF15 nuclear levels, we conducted immunofluorescence assays in cells transfected with pGFP-GDF15 (Fig. 3). In control cells not exposed to fatty acids or those incubated with oleate, GDF15 seemed to be mainly present in the nucleus, specifically in the nucleolus, which agrees with previous reports [15]. However, in palmitate-exposed cells, nuclear GDF15 showed an intense reduction in GDF15 levels, whereas the content of this protein was increased in cytoplasm. Finally, incubation of palmitate-exposed cells with leptomycin B prevented the reduction in nuclear GDF15 levels caused by the saturated fatty acid.

### The reduction in nuclear GDF15 levels caused by palmitate increases SMAD3 and PAI-1 protein levels in human LHCN-M2 myotubes

**Fig. 4:**
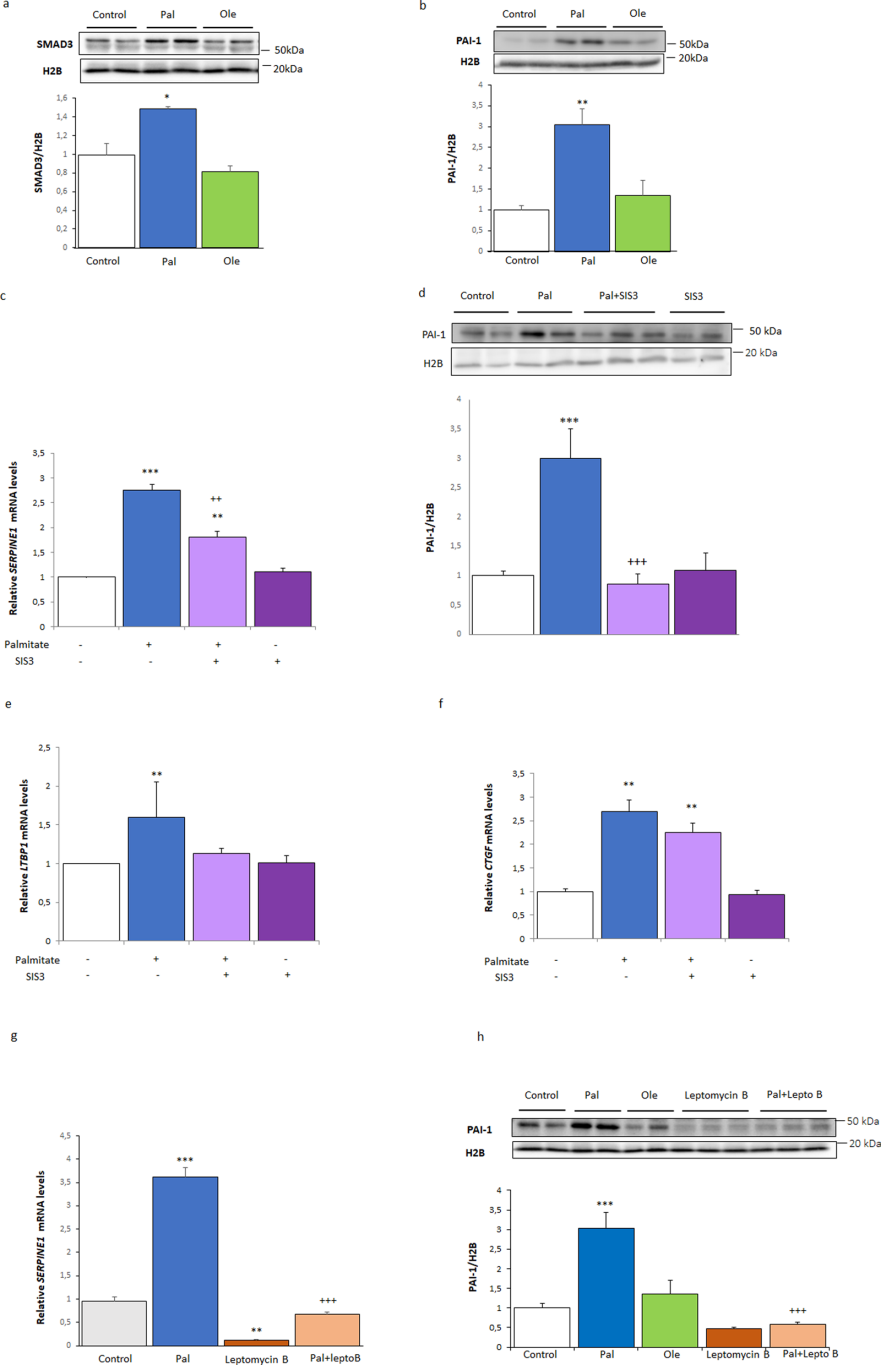
Palmitate, but not oleate, increases SMAD3 and PAI-1 levels in human LHCN-M2 myotubes. Immunoblot analysis of **a** SMAD3 and **b** PAI-1 in nuclear extracts from human LHCN-M2 myotubes incubated in the presence or absence (control) of 0.5 mM palmitate (Pal) or 0.5 mM oleate (Ole) for 16 h (n = 4). **c** *SERPINE1* mRNA levels (n = 3) and immunoblot analysis of **d** PAI-1 (n = 4) in human LHCN-M2 myotubes incubated for 16 h in the presence or absence (control) of 0.5 mM palmitate (Pal) with or without 10 µM SIS3. **e** *LTBP1* and **f** *CTGF* mRNA levels in human LHCN-M2 myotubes incubated for 16 h in the presence or absence (control) of 0.5 mM palmitate (Pal) with or without 10 µM SIS3 (n = 3). **g** *SERPINE1* mRNA levels and immunoblot analysis of **h** PAI-1 in nuclear extracts from human LHCN-M2 myotubes incubated in the presence or absence (control) of 0.5 mM palmitate (Pal) with or without 10 nM leptomycin B or 0.5 mM oleate (Ole) for 16 h (n = 3). Data are presented as the mean ± SEM. *p < 0.05, **p < 0.01, and ***p < 0.001 *versus* control. ^++^*p* < 0.01, and ^+++^*p* < 0.001 *vs.* palmitate-treated cells

As reported previously, nuclear GDF15 reduces the DNA binding capacity of the SMAD complex, leading to an attenuation of TGF-β1-induced SMAD signaling [2, 15]. Next, we examined the protein levels of SMAD3 and PAI-1, encoded by the SMAD3 target gene *SERPINE1*. Consistent with the changes in nuclear GDF15 levels, palmitate, but not oleate, increased the nuclear protein levels of both SMAD3 and PAI-1 (Fig. 4a, b). To confirm that the increase in SERPINE1/PAI-1 levels caused by palmitate was mediated by SMAD3, a critical intracellular mediator of TGF-β1 signaling that is regulated by phosphorylation, we used the specific inhibitor of SMAD3 phosphorylation, SIS3 [29]. Incubation of the cells with palmitate increased *SERPINE1* mRNA and PAI-1 protein levels, with these changes prevented or attenuated when the cells were co-incubated with both palmitate and SIS3 (Fig. 4c, d). Similarly, the palmitate-mediated increase in the expression of another SMAD3 target gene, latent transforming growth factor-beta binding protein 1 (*LTBP1*), was abolished by SIS3, although that of connective tissue growth factor (*CTGF*) was not significantly reduced (Fig. 4e, f). In agreement with a role for the nuclear export of GDF15 to the cytosol in the derepression/activation of the SMAD3-PAI-1 pathway, treatment with leptomycin B completely dampened the increase in the mRNA levels of *SERPINE1* after 16 (Fig. 4g) or 48 h (Supplementary Fig. 1b) and, consistently, abrogated the increase in PAI-1 protein levels (Fig. 4h).

### *Gdf15*-knockout mice fed an HFD show increased glucose intolerance and activation of the SMAD3-PAI-1 pathway in their skeletal muscle

**Fig. 5:**
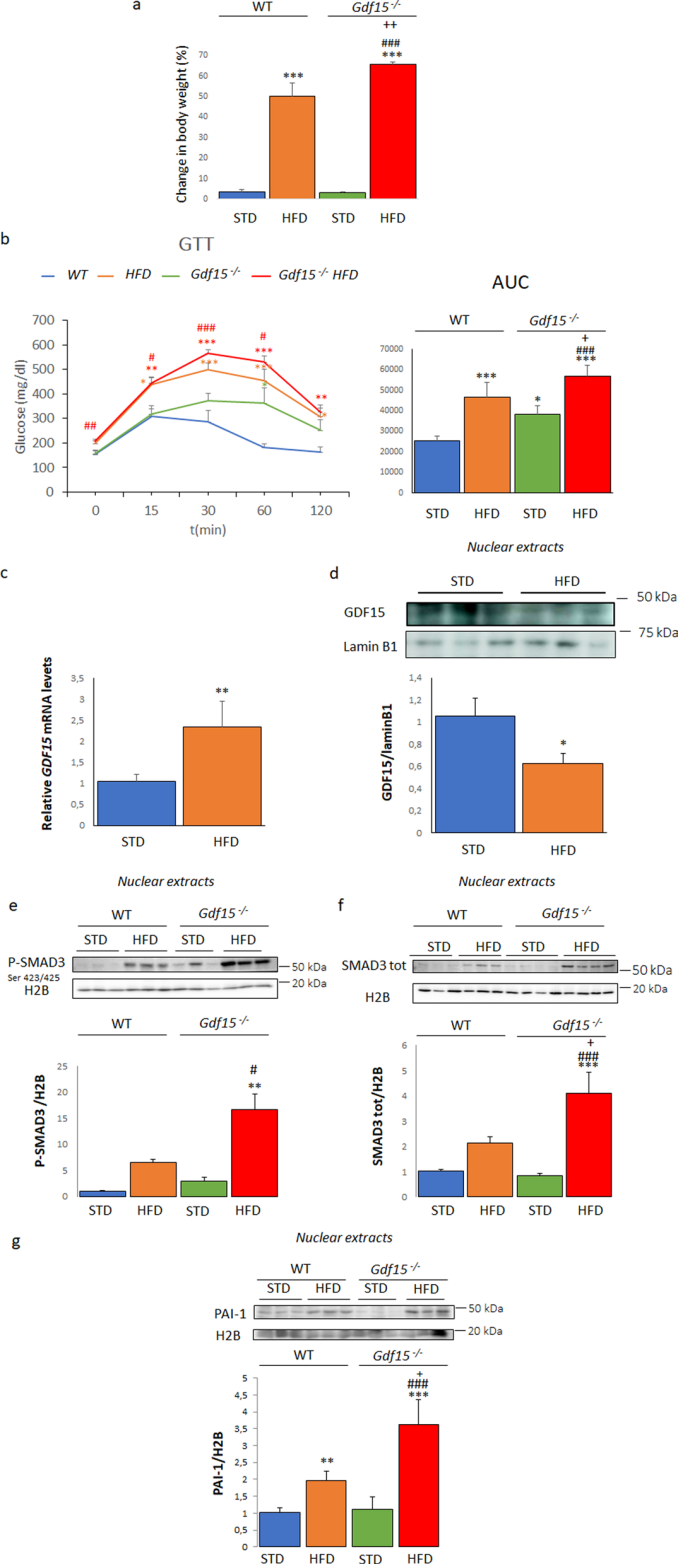
HFD-fed *Gdf15*^−/−^ mice display exacerbated glucose intolerance and increased levels of SMAD3 and PAI-1 in their skeletal muscle compared to HFD-fed WT mice. **a** Change in body weight in *Gdf15*^−/−^ and wild-type (WT) littermates fed a standard diet (STD) or a high-fat diet (HFD) for 16 weeks (n = 6). **b** Glucose tolerance test and area under the curve (AUC) (n = 5). **c** mRNA (n = 4) and **d** protein GDF15 (n = 5) levels in WT mice fed an STD or HFD. Immunoblot analysis of **e** phosphorylated (n = 3) and **f** total SMAD3 (n = 3–4) and **g** PAI-1 (n = 5) in the skeletal muscle of *Gdf15*^−/−^ and wild-type (WT) littermates fed an STD or HFD for 16 weeks. Data are presented as the mean ± SEM. *p < 0.05, **p < 0.01, and ***p < 0.001 *versus* STD-fed WT mice. ^#^*p* < 0.05, ^##^*p* < 0.01, and ^###^*p* < 0.001 *versus* STD-fed *Gdf15*^−/−^ mice. ^+^*p* < 0.05 *versus* HFD-fed WT mice

**Fig. 6:**
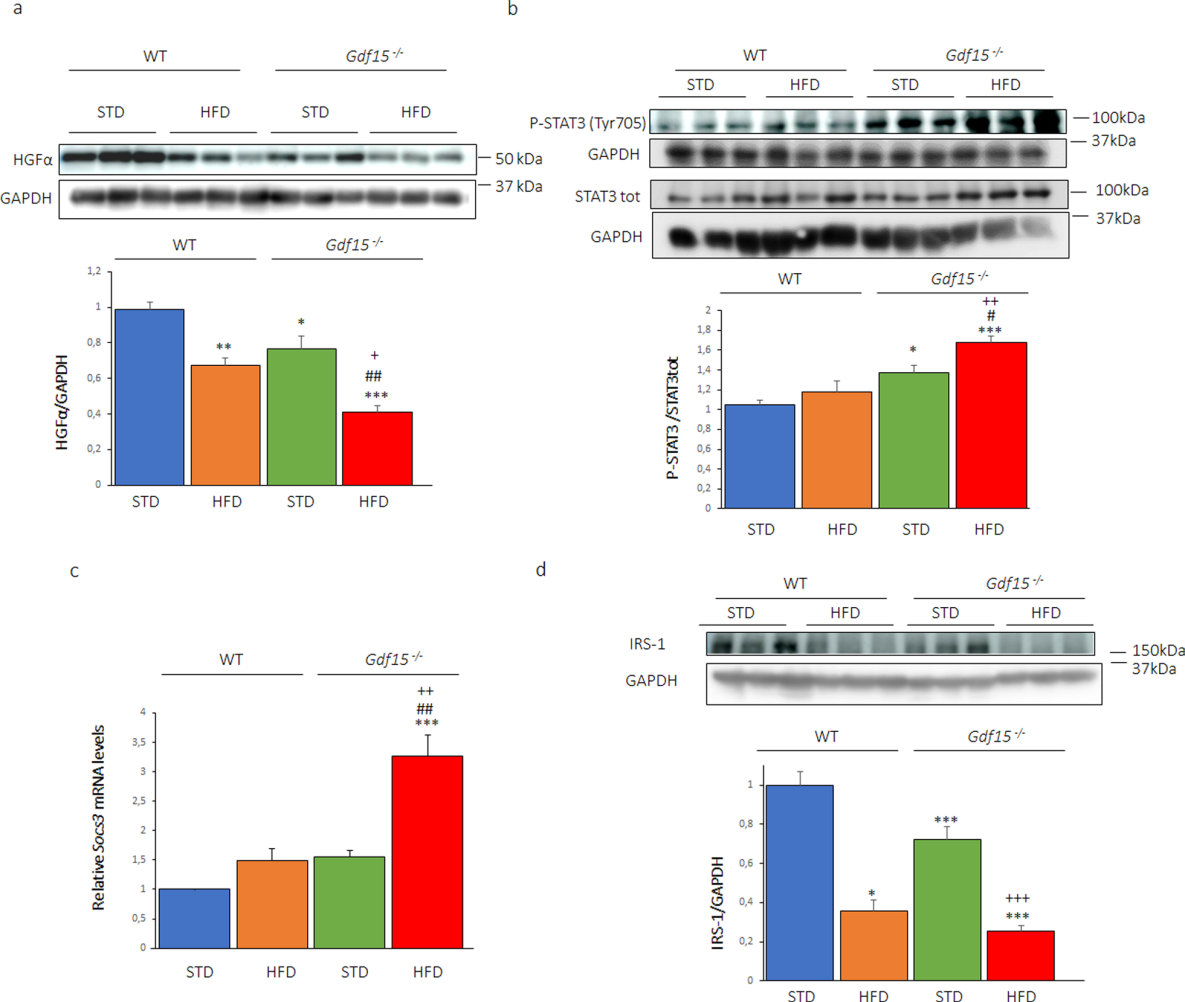
HFD-fed *Gdf15*^−/−^ mice display reduced levels of HGFα and increased activation of the STAT3-SOCS3 pathway in their skeletal muscle compared to HFD-fed WT mice. **a** Immunoblot analysis of HGFα in the skeletal muscle of *Gdf15*^−/−^ and wild-type (WT) littermates fed a standard diet (STD) or a high-fat diet (HFD) for 16 weeks (n = 4). **b** Immunoblot analysis of total and phosphorylated STAT3 in skeletal muscle (n = 5). **c** *SOCS3* mRNA levels in *Gdf15*^−/−^ and wild-type (WT) littermates fed an STD or HFD (n = 3). **d** Immunoblot analysis of IRS1 in skeletal muscle (n = 3). Data are presented as the mean ± SEM. *p < 0.05, **p < 0.01, and ***p < 0.001 *versus* STD-fed WT mice. ^#^*p* < 0.05 and ^##^*p* < 0.01 *versus* STD-fed *Gdf15*^−/−^ mice. ^+^*p* < 0.05, ^++^*p* < 0.01 and ^+++^*p* < 0.001 *versus* HFD-fed WT mice

To evaluate whether nuclear GDF15 is implicated in the SMAD3-PAI-1 pathway in vivo, we used WT and *Gdf15*^−/−^ mice. Animals fed the HFD showed an increase in body weight compared to those fed the STD, with the increase significantly higher for HFD-fed *Gdf15*^−/−^ mice (Fig. 5a). *Gdf15*^−/−^ mice displayed glucose intolerance compared to WT mice and feeding these mice the HFD resulted in a significant worsening of glucose intolerance compared to WT mice fed the same diet (Fig. 5b). In addition, the HFD increased *Gdf15* mRNA levels (Fig. 5c), whereas it reduced the nuclear levels of GDF15 in the skeletal muscle of WT mice (Fig. 5d). When we examined the SMAD3-PAI-1 pathway in the skeletal muscle, we observed that the HFD significantly increased the nuclear levels of total (Fig. 5e) and phosphorylated SMAD3 (Fig. 5f) in WT mice, with these increases exacerbated in the HFD-fed *Gdf15*^−/−^ mice. Consistent with these changes, PAI-1 showed the same trend of an increase in HFD-fed WT mice that was aggravated in the *Gdf15*^−/−^ mice fed the same diet (Fig. 5g). Interestingly, PAI-1 has been reported to reduce the amount of active HGF [30], a cytokine that increases glucose transport ex vivo in the skeletal muscle [31] and whose overexpression in this tissue counteracts muscle insulin resistance and improves glucose tolerance in an animal model of obesity [32]. In agreement with the changes in PAI-1, the protein levels of HGFα were reduced in HFD-fed WT mice, with this effect significantly exacerbated in the *Gdf15*^−/−^ mice fed the same diet (Fig. 6a). Moreover, PAI-1 has been described as a key ligand of signal transducer and activator of transcription 3 (STAT3) in a mouse model of peritoneal carcinomatosis [33]. STAT3 upregulates the expression of suppressor of cytokine signaling 3 (SOCS3), which inhibits insulin signaling through several distinct mechanisms, including by directly interfering with insulin receptor activation, blocking IRS activation, and inducing IRS degradation [34]. Dimerization, nuclear translocation, and an increase in the transcriptional activity of STAT3 require its phosphorylation at Tyr^705^. STD-fed *Gdf15*^−/−^ mice showed a slight increase in the phosphorylation of STAT3 at Tyr^705^ in their skeletal muscle that was exacerbated in the HFD-fed *Gdf15*^−/−^ mice (Fig. 6b). Accordingly, the expression levels of *Socs3* were only significantly upregulated in the skeletal muscle of HFD-fed *Gdf15*^−/−^ mice (Fig. 6c). Next, we examined the protein levels of IRS-1 in the skeletal muscle. HFD-fed WT mice and STD-fed *Gdf15*^−/−^ mice showed reduced IRS-1 protein levels that were aggravated in the HFD-fed *Gdf15*^−/−^ mice (Fig. 6d). These findings show that the increase in PAI-1 levels in HFD-fed *Gdf15*^−/−^ mice is accompanied by a reduction in HGFα levels and an overactivation of the STAT3-SOCS3 pathway. The activation of these pathways in the context of GDF15 deficiency and high lipid content may have a strong impact on insulin signaling, thereby contributing to insulin resistance.

### GDF15 nuclear export inhibition moderates the decline in insulin-stimulated Akt phosphorylation caused by palmitate in human LHCN-M2 myotubes

**Fig. 7:**
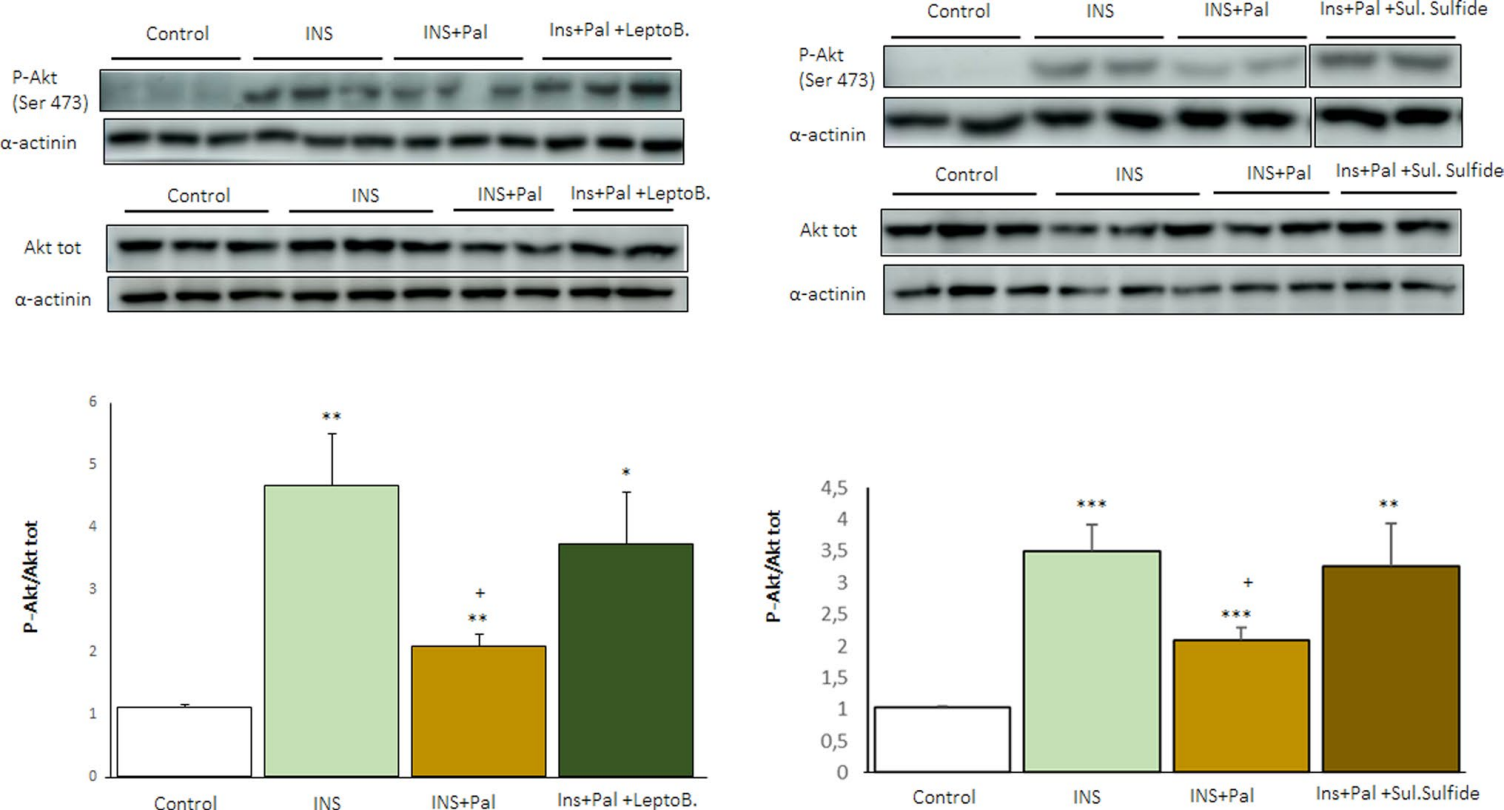
Increasing GDF15 nuclear levels moderates the decline in insulin-stimulated Akt phosphorylation caused by palmitate exposure in human LHCN-M2 myotubes. Immunoblot analysis of total and phosphorylated Akt in human LHCN-M2 myotubes incubated for 16 h in the presence or absence (control) of 0.5 mM palmitate (Pal), 5 nM leptomycin B or 10 µM sulindac sulfide. When indicated, LHCN‑M2 myotubes were incubated with or without 100 nM of insulin (Ins) for the last 10 min. (n = 4). Fragments of the same original image were spliced together to remove irrelevant lanes (Supplementary Fig. 2). Data are presented as the mean ± SEM. *p < 0.05, **p < 0.01, and ***p < 0.001 *versus* control. ^+^*p* < 0.05 *versus* insulin-stimulated cells

Finally, to demonstrate that GDF15 nuclear export influences the insulin signaling pathway, LHCN-M2 myotubes were incubated with the inhibitor of GDF15 nuclear export leptomycin B in the presence or absence of palmitate and stimulated with insulin or vehicle. In addition, cells were incubated with sulindac sulfate, a nonsteroidal anti-inflammatory drug reported to increase GDF15 nuclear levels [15]. As expected, insulin stimulated Akt phosphorylation, whereas palmitate exposure attenuated this increase (Fig. 7). However, the effect of palmitate was diminished in the presence of the nuclear export inhibitor leptomycin B. Likewise, treatment with sulindac sulfate prevented the reduction of Akt phosphorylation by palmitate (Fig. 7). Overall, these findings suggest a link between GDF15 nuclear levels and the occurrence of insulin resistance.

## Discussion

**Fig. 8:**
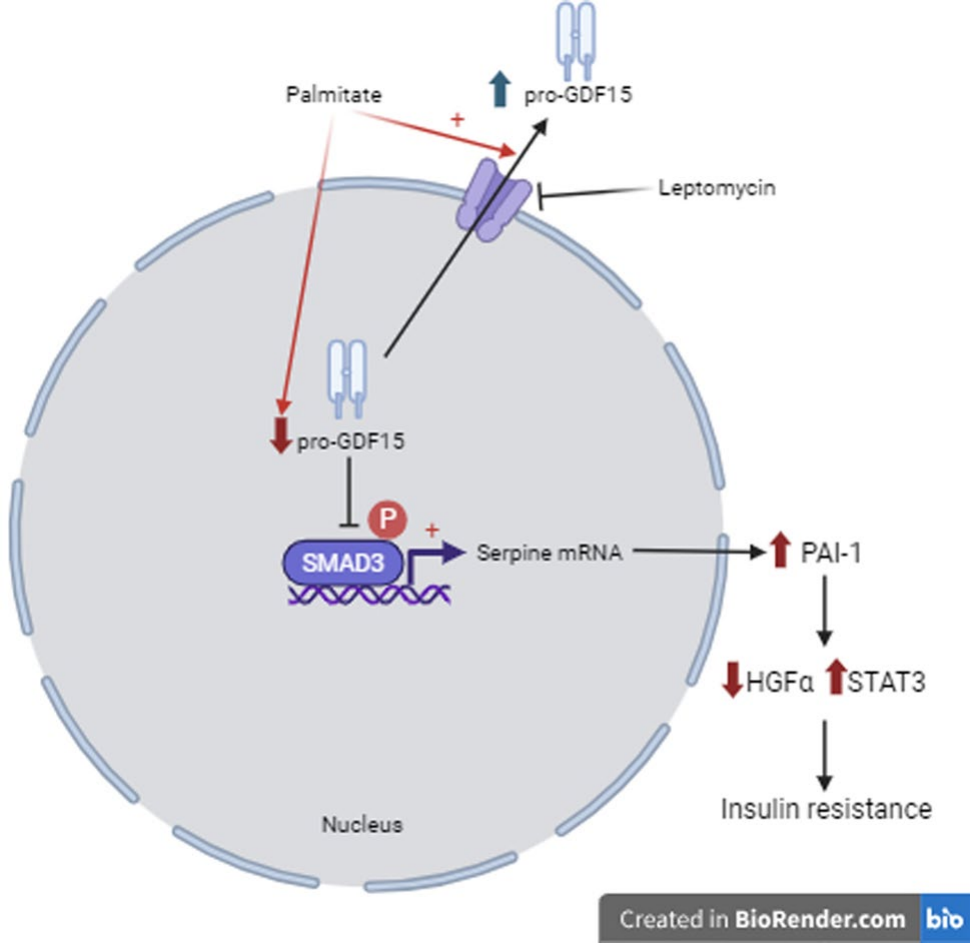
Palmitate, but not oleate, reduces the nuclear levels of GDF15. This reduction is accompanied by an increase in the protein levels of SMAD3 and of its target SERPINE1/PAI-1. These changes caused by palmitate are prevented by the nuclear export inhibitor leptomycin B. The increase in PAI-1 results in changes in its targets HGFα and STAT3-SOCS3, leading to the development of insulin resistance

GDF15 is a regulator of food intake, as well as glucose and lipid metabolism [3]. This stress cytokine reduces food intake and body weight by activating the central receptor GFRAL located in the hindbrain-brainstem. Although many of the beneficial effects of GDF15 on glucose homeostasis are mediated by the reduction in food intake and body weight, several lines of evidence indicate that GDF15 may also have peripheral effects [11, 13, 14]. Interestingly, in the cell nucleus, GDF15 inhibits the binding of the SMAD complex to its response elements in the promoters of its target genes, thus attenuating TGF-β1-induced SMAD signaling and affecting cell migration and invasion [2, 15]. However, whether this nuclear action of GDF15 impacts metabolism has remained unexplored. Here, we show that the saturated FA palmitate increases the expression of GDF15 in human myotubes. However, at the same time, the nuclear levels of GDF15 are reduced by this saturated FA. This reduction is prevented by the nuclear export inhibitor leptomycin B and is not observed in the cells incubated with the monounsaturated FA oleate. The reduction in nuclear GDF15 levels caused by palmitate is accompanied by an increase in the levels of SMAD3 and the expression of its target genes, pointing to an activation of the SMAD3 pathway by palmitate, but not by oleate. Furthermore, HFD-fed *Gdf15*^−/−^ mice display aggravated glucose intolerance compared to HFD-fed WT mice. In the skeletal muscle, there are increased levels of phosphorylated SMAD3 and of its target gene *SERPINE1* that encodes PAI-1. The increase in PAI-1 levels in the skeletal muscle of HFD-fed *Gdf15*^−/−^ mice is accompanied by changes in its targets HGFα and STAT3-SOCS3, leading to the development of insulin resistance (Fig. 8). Overall, these findings suggest that the saturated FA palmitate activates the SMAD3-PAI-1 pathway by reducing the nuclear levels of GDF15, a mechanism that is reported for the first time here.

The saturated fatty acid palmitate and the monounsaturated fatty acid oleate were selected in this study because they are the most abundant dietary and plasma fatty acids [19, 35]. Their behavior is completely different in the development of insulin resistance. Whereas palmitate promotes insulin resistance, oleate has a protective role on this condition [19]. These fatty acids also differ in their capacity to induce the integrated stress response (ISR), one of the best-characterized pathways involved in the upregulation of GDF15. Activation of the UPR by saturated FAs increases GDF15 expression [36]. For example, the saturated FA palmitate has been reported to activate the UPR and induce the expression of *GDF15* through CHOP in THP-1 cells and macrophages [20]. In fact, CHOP directly binds to the *GDF15* promoter region and regulates GDF15 expression. Consistent with this, palmitate increases the levels of both GDF15 and CHOP in human LHCN-M2 myotubes, while the monounsaturated FA oleate does not. Moreover, co-incubation of palmitate with a lower oleate concentration attenuates the increases in CHOP and GDF15 levels. These findings agree with those of previous studies reporting that palmitate activates the UPR, whereas oleate does not, and that oleate can alleviate the effects of palmitate on the UPR (for a review see [19]). The increase in *GDF15* expression caused by palmitate is accompanied by the upregulation of GDF15 protein levels in the cytosol. Surprisingly, the nuclear levels of GDF15 are reduced by palmitate. Since it has been reported that nuclear GDF15 interacts with CRM1 for export out of the nucleus [2, 15], we used a CRM1 inhibitor to revert the effect of palmitate. Our findings show that the CRM1 inhibitor leptomycin B reverses the reduction in nuclear GDF15 levels caused by palmitate. Moreover, as mentioned above, a previous study reported that pro-GDF15 accumulates in the nucleus, attenuating TGF-β1-induced signaling by interrupting the DNA binding capacity of the SMAD complex [15]. How palmitate regulates GDF15 nuclear levels has not been explored in this study. It has been previously reported that the transcription factor EB (TFEB) nuclear export is mediated by CRM1, and is modulated by nutrient availability via mammalian target of rapamycin (mTOR)-dependent hierarchical multisite phosphorylation of Ser^142^ and Ser^138^ in C2C12 myotubes [37]. Since palmitate, but not oleate, activates the mTOR signaling [38], this pathway might underneath under the nuclear regulation of GDF15 by this saturated fatty acid. This possibility should be explored in future studies.

Consistent with the role of nuclear GDF15 as an inhibitor of the transcriptional activity of SMADs, the reduction in nuclear GDF15 levels caused by palmitate is accompanied by an increase in the protein levels of SMAD3 and of its target *SERPINE1*/PAI-1. It is worth mentioning that SMAD3 levels are elevated in primary human skeletal muscle cells derived from obese females [39]. Silencing SMAD3 in these cells improves insulin-stimulated AS160 phosphorylation and glucose uptake [39]. However, it is unclear how the increase in SMAD3 leads to the attenuation of insulin signaling. The findings of our study may provide new determinants by which the increase in SMAD3 levels can contribute to the development of insulin resistance in skeletal muscle. Herein, we show that *SERPINE1*/PAI-1, a SMAD3 target, is upregulated by palmitate in human myotubes. In addition, HFD-fed *Gdf15*^−/−^ mice show increased levels of phosphorylated SMAD3 and *SERPINE1*/PAI-1 in their skeletal muscle compared to HFD-fed WT mice. PAI-1 is an acute-phase protein and antifibrinolytic agent that is consistently up-regulated in obesity and linked to elevated cardiovascular anomalies in obese patients [40]. However, little is known about the role of PAI-1 in insulin resistance. Remarkably, excess hepatic PAI-1 in the context of obesity-induced T2DM disrupts HGF activation in the liver, leading to impaired signaling through its receptor that is encoded by the gene *MET* [30]. Consistent with this, when we examined the levels of HGFα, we observed that, in agreement with the highest levels of PAI-1, HFD-fed *Gdf15*^−/−^ mice showed the most intense reduction in HGFα levels in their skeletal muscle. The reduction in HGFα levels in the context of skeletal muscle may result in impaired glucose metabolism since HGF-targeted delivery to the skeletal muscle improves glucose homeostasis in mice with diet-induced obesity [32].

We also examined whether the increase in SMAD3-PAI-1 could mediate insulin resistance through a different mechanism. In peritoneal carcinomatosis, PAI-1 acts as a STAT3 ligand [33], but it is unknown whether this mechanism operates in skeletal muscle. We observed that the increase in PAI-1 levels in the skeletal muscle of HFD-fed *Gdf15*^−/−^ mice was accompanied by the activation of the STAT3-SOCS3 pathway. Moreover, since the STAT3-SOCS3 pathway attenuates insulin signaling through several mechanisms, including IRS-1 degradation [34], we observed a reduction in IRS-1 protein levels that was aggravated in the HFD-fed *Gdf15*^−/−^ mice.

This study also contributes to a more comprehensive understanding of the effects of saturated and monounsaturated FA on insulin signaling, underlining the relevance of the activation of the SMAD3-PAI-1 pathway by FAs. Our findings demonstrate that palmitate reduces nuclear GDF15 levels and increases the levels of SMAD3 and PAI-1 in human myotubes, while oleate does not. As a result of the increase in PAI-1 levels caused by palmitate, this FA may promote additional mechanisms such as a reduction in HGFα levels and the activation of the STAT3-SOCS3 pathway, thereby attenuating insulin signaling. Additional studies are needed to confirm the role of GDF15 nuclear translocation and the subsequent changes in the SMAD3-PAI-1 pathway in the insulin signaling pathway. More specifically, the use of muscle-specific *Gdf15* knockout mice might help to decipher potential new intricated mechanisms involved in the development of skeletal muscle insulin resistance. Likewise, although studying GDF15 in the seletal muscle is relevant since it is the tissue where most insulin-stimulated glucose use occurs in the whole organism [24, 25], and GDF15 enhances energy expenditure in this tissue muscle [41], GDF15 is also expressed at high levels in critical tissues for the development of insulin resistance, such as the liver and adipose tissue. It remains to evaluate if palmitate-mediated regulation of GDF15 nuclear levels is also observed in these tissues.

Overall, the findings of this study uncover a new mechanism by which the saturated FA palmitate activates the SMAD3-PAI-1 pathway in skeletal muscle. By reducing the nuclear levels of GDF15, palmitate increases SMAD3 levels and upregulates the levels of its target genes, including *SERPINE1*/PAI-1. The increase in PAI-1 levels is accompanied by a reduction in HGFα levels and the activation of the STAT3-SOCS3 pathway in the skeletal muscle of HFD-fed *Gdf15*^−/−^ mice. Given the proposed role of the SMAD3-PA-I pathway in insulin resistance, these data suggest that deciphering the mechanisms involved in regulation of nuclear GDF15 levels may evolve into a new strategy to prevent the development of insulin resistance and T2DM.

## Supplementary Information

Below is the link to the electronic supplementary material.

Supplementary file1 (TIF 2465 KB)

Supplementary file2 (PDF 31 KB)

## Data Availability

The study data are available on demand.

## Abbreviations

ATF4: Activating transcription factor 4
CHOP: C/EBP homologous protein
CRM1: Chromosome region maintenance 1
CTGF: Connective tissue growth factor
ER: Endoplasmic reticulum
FA: Fatty acid
GDF15: Growth differentiation factor 15
GFRAL: Glial cell line-derived neurotrophic factor (GDNF)-family receptor α-like
GTT: Glucose tolerance test
HFD: High-fat diet
HGF: Hepatocyte growth factor
InsR: Insulin receptor
IRS: Insulin receptor substrate
ISR: Integrated stress response
LTBP1: Latent transforming growth factor beta binding protein 1
PAI-1: Plasminogen activator inhibitor 1
PPAR: Peroxisome proliferator-activated receptor
SOCS3: Suppressor of cytokine signaling 3
STD: Standard diet
STAT3: Signal transducer and activator of transcription 3
TGF-β: Transforming growth factor β
T2DM: Type 2 diabetes mellitus
UPR: Unfolded protein response

## References

[1] Aguilar-Recarte D et al (2022) Knocking on GDF15’s door for the treatment of type 2 diabetes mellitus. Trends Endocrinol Metab 33:741–754. 10.1016/j.tem.2022.08.004 36151002 10.1016/j.tem.2022.08.004

[2] Baek SJ, Eling T (2019) Growth differentiation factor 15 (GDF15): a survival protein with therapeutic potential in metabolic diseases. Pharmacol Ther 198:46–58. 10.1016/j.pharmthera.2019.02.008 30790643 10.1016/j.pharmthera.2019.02.008 PMC7196666

[3] Patel S et al (2019) GDF15 provides an endocrine signal of nutritional stress in mice and humans. Cell Metab 29(707–718):e708. 10.1016/j.cmet.2018.12.016 10.1016/j.cmet.2018.12.016 PMC6408327 30639358

[4] Ji X et al (2017) Growth differentiation factor 15 is a novel diagnostic biomarker of mitochondrial diseases. Mol Neurobiol 54:8110–8116. 10.1007/s12035-016-0283-7 27889897 10.1007/s12035-016-0283-7

[5] Mullican SE et al (2017) GFRAL is the receptor for GDF15 and the ligand promotes weight loss in mice and nonhuman primates. Nat Med 23:1150–1157. 10.1038/nm.4392 28846097 10.1038/nm.4392

[6] Hsu JY et al (2017) Non-homeostatic body weight regulation through a brainstem-restricted receptor for GDF15. Nature 550:255–259. 10.1038/nature24042 28953886 10.1038/nature24042

[7] Yang L et al (2017) GFRAL is the receptor for GDF15 and is required for the anti-obesity effects of the ligand. Nat Med 23:1158–1166. 10.1038/nm.4394 28846099 10.1038/nm.4394

[8] Emmerson PJ et al (2017) The metabolic effects of GDF15 are mediated by the orphan receptor GFRAL. Nat Med 23:1215–1219. 10.1038/nm.4393 28846098 10.1038/nm.4393

[9] Borner T et al (2020) GDF15 induces anorexia through nausea and emesis. Cell Metab 31(351–362):e355. 10.1016/j.cmet.2019.12.004 10.1016/j.cmet.2019.12.004 PMC7161938 31928886

[10] Borner T et al (2020) GDF15 induces an aversive visceral malaise state that drives anorexia and weight loss. Cell Rep 31:107543. 10.1016/j.celrep.2020.107543 32320650 10.1016/j.celrep.2020.107543 PMC7271892

[11] Aguilar-Recarte D et al (2021) GDF15 mediates the metabolic effects of PPARbeta/delta by activating AMPK. Cell Rep 36:109501. 10.1016/j.celrep.2021.109501 34380027 10.1016/j.celrep.2021.109501

[12] Aguilar-Recarte D et al (2022) A positive feedback loop between AMPK and GDF15 promotes metformin antidiabetic effects. Pharmacol Res 187:106578. 10.1016/j.phrs.2022.106578 36435271 10.1016/j.phrs.2022.106578

[13] Weng JH et al (2021) Colchicine acts selectively in the liver to induce hepatokines that inhibit myeloid cell activation. Nat Metab 3:513–522. 10.1038/s42255-021-00366-y 33846641 10.1038/s42255-021-00366-y PMC8175070

[14] Wang Z et al (2021) GDF15 induces immunosuppression via CD48 on regulatory T cells in hepatocellular carcinoma. J Immunother Cancer 9. 10.1136/jitc-2021-002787 10.1136/jitc-2021-002787 PMC8422483 34489334

[15] Min KW et al (2016) NAG-1/GDF15 accumulates in the nucleus and modulates transcriptional regulation of the Smad pathway. Oncogene 35:377–388. 10.1038/onc.2015.95 25893289 10.1038/onc.2015.95 PMC4613816

[16] Nyarady BB et al (2024) Growth and differentiation factor-15: a link between inflammaging and cardiovascular disease. Biomed Pharmacother 174:116475. 10.1016/j.biopha.2024.116475 38522236 10.1016/j.biopha.2024.116475

[17] Lockhart SM et al (2020) GDF15: a hormone conveying somatic distress to the brain. Endocr Rev 41. 10.1210/endrev/bnaa007 10.1210/endrev/bnaa007 PMC7299427 32310257

[18] Salvado L et al (2015) Targeting endoplasmic reticulum stress in insulin resistance. Trends Endocrinol Metab 26:438–448. 10.1016/j.tem.2015.05.007 26078196 10.1016/j.tem.2015.05.007

[19] Palomer X et al (2018) Palmitic and oleic acid: the Yin and Yang of fatty acids in type 2 diabetes mellitus. Trends Endocrinol Metab 29:178–190. 10.1016/j.tem.2017.11.009 29290500 10.1016/j.tem.2017.11.009

[20] L’Homme L et al (2020) Saturated fatty acids promote GDF15 expression in human macrophages through the PERK/eIF2/CHOP signaling pathway. Nutrients 12. 10.3390/nu12123771 10.3390/nu12123771 PMC7764024 33302552

[21] Kim J et al (2021) TFEB-GDF15 axis protects against obesity and insulin resistance as a lysosomal stress response. Nat Metab 3:410–427. 10.1038/s42255-021-00368-w 33758420 10.1038/s42255-021-00368-w

[22] Montori-Grau M et al (2022) Endoplasmic reticulum stress downregulates PGC-1alpha in skeletal muscle through ATF4 and an mTOR-mediated reduction of CRTC2. Cell Commun Signal 20:53. 10.1186/s12964-022-00865-9 35428325 10.1186/s12964-022-00865-9 PMC9012021

[23] Salvado L et al (2013) Oleate prevents saturated-fatty-acid-induced ER stress, inflammation and insulin resistance in skeletal muscle cells through an AMPK-dependent mechanism. Diabetologia 56:1372–1382. 10.1007/s00125-013-2867-3 23460021 10.1007/s00125-013-2867-3

[24] DeFronzo RA et al (1985) Effects of insulin on peripheral and splanchnic glucose metabolism in noninsulin-dependent (type II) diabetes mellitus. J Clin Invest 76:149–155. 10.1172/JCI111938 3894418 10.1172/JCI111938 PMC423730

[25] DeFronzo RA et al (1981) The effect of insulin on the disposal of intravenous glucose. Results from indirect calorimetry and hepatic and femoral venous catheterization. Diabetes 30:1000–1007. 10.2337/diab.30.12.1000 7030826 10.2337/diab.30.12.1000

[26] Baque S et al (1996) Overexpression of muscle glycogen phosphorylase in cultured human muscle fibers causes increased glucose consumption and nonoxidative disposal. J Biol Chem 271:2594–2598. 10.1074/jbc.271.5.2594 8576226 10.1074/jbc.271.5.2594

[27] Zhu CH et al (2007) Cellular senescence in human myoblasts is overcome by human telomerase reverse transcriptase and cyclin-dependent kinase 4: consequences in aging muscle and therapeutic strategies for muscular dystrophies. Aging Cell 6:515–523. 10.1111/j.1474-9726.2007.00306.x 17559502 10.1111/j.1474-9726.2007.00306.x

[28] Dimauro I et al (2012) A simple protocol for the subcellular fractionation of skeletal muscle cells and tissue. BMC Res Notes 5:513. 10.1186/1756-0500-5-513 22994964 10.1186/1756-0500-5-513 PMC3508861

[29] Ji X et al (2018) Specific Inhibitor of Smad3 (SIS3) attenuates fibrosis, apoptosis, and inflammation in unilateral ureteral obstruction kidneys by inhibition of transforming growth factor beta (TGF-beta)/Smad3 signaling. Med Sci Monit 24:1633–1641. 10.12659/msm.909236 10.12659/MSM.909236 PMC5872904 29555895

[30] Coudriet GM et al (2019) A noncanonical role for plasminogen activator inhibitor type 1 in obesity-induced diabetes. Am J Pathol 189:1413–1422. 10.1016/j.ajpath.2019.04.004 31054988 10.1016/j.ajpath.2019.04.004

[31] Perdomo G et al (2008) Hepatocyte growth factor is a novel stimulator of glucose uptake and metabolism in skeletal muscle cells. J Biol Chem 283:13700–13706. 10.1074/jbc.M707551200 18362143 10.1074/jbc.M707551200 PMC2376221

[32] Sanchez-Encinales V et al (2015) Targeted delivery of HGF to the skeletal muscle improves glucose homeostasis in diet-induced obese mice. J Physiol Biochem 71:795–805. 10.1007/s13105-015-0444-6 26507644 10.1007/s13105-015-0444-6

[33] Hendrikson J et al (2022) Ligand-mediated PAI-1 inhibition in a mouse model of peritoneal carcinomatosis. Cell Rep Med 3:100526. 10.1016/j.xcrm.2022.100526 35243423 10.1016/j.xcrm.2022.100526 PMC8861959

[34] Howard JK, Flier JS (2006) Attenuation of leptin and insulin signaling by SOCS proteins. Trends Endocrinol Metab 17:365–371. 10.1016/j.tem.2006.09.007 17010638 10.1016/j.tem.2006.09.007

[35] Buchanan CDC et al (2021) Analysis of major fatty acids from matched plasma and serum samples reveals highly comparable absolute and relative levels. Prostaglandins Leukot Essent Fatty Acids 168:102268. 10.1016/j.plefa.2021.102268 33831721 10.1016/j.plefa.2021.102268

[36] Wang D et al (2024) Fatty acids increase GDF15 and reduce food intake through a GFRAL signaling axis. Diabetes 73:51–56. 10.2337/db23-0495 37847913 10.2337/db23-0495 PMC10784653

[37] Napolitano G et al (2018) mTOR-dependent phosphorylation controls TFEB nuclear export. Nat Commun 9:3312. 10.1038/s41467-018-05862-6 30120233 10.1038/s41467-018-05862-6 PMC6098152

[38] Kwon B, Querfurth HW (2015) Palmitate activates mTOR/p70S6K through AMPK inhibition and hypophosphorylation of raptor in skeletal muscle cells: reversal by oleate is similar to metformin. Biochimie 118:141–150. 10.1016/j.biochi.2015.09.006 26344902 10.1016/j.biochi.2015.09.006

[39] Saxena G et al (2024) Sex-specific increases in myostatin and SMAD3 contribute to obesity-related insulin resistance in human skeletal muscle and primary human myotubes. Am J Physiol Endocrinol Metab 326:E352–E365. 10.1152/ajpendo.00199.2023 38088865 10.1152/ajpendo.00199.2023 PMC11193514

[40] Plomgaard P et al (1985) (2005) TNF-alpha, but not IL-6, stimulates plasminogen activator inhibitor-1 expression in human subcutaneous adipose tissue. J Appl Physiol 98:2019–2023. 10.1152/japplphysiol.01220.2004 10.1152/japplphysiol.01220.2004 15677734

[41] Wang D et al (2023) GDF15 promotes weight loss by enhancing energy expenditure in muscle. Nature 619:143–150. 10.1038/s41586-023-06249-4 37380764 10.1038/s41586-023-06249-4 PMC10322716

