# Supplementary figures and images for: Palmitate potentiates the SMAD3-PAI-1 pathway by reducing nuclear GDF15 levels
Source: Cell Mol Life Sci. 2025 Jan 18;82(1):43. doi: 10.1007/s00018-024-05571-y (PMC11741968; doi:10.1007/s00018-024-05571-y)

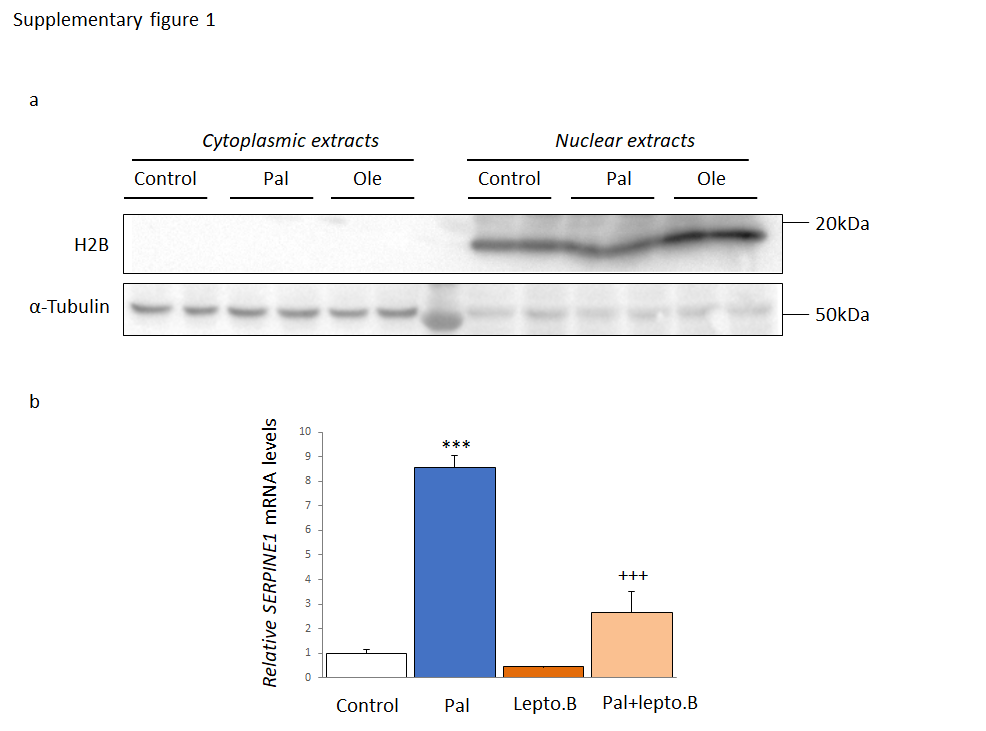

Supplement: Supplementary file 1 — Supplementary file1 (TIF 2465 KB) [file 18_2024_5571_MOESM1_ESM.tif]
