# Supplementary material for: Palmitate potentiates the SMAD3-PAI-1 pathway by reducing nuclear GDF15 levels
Source: Cell Mol Life Sci. 2025 Jan 18;82(1):43. doi: 10.1007/s00018-024-05571-y (PMC11741968; doi:10.1007/s00018-024-05571-y)

In blot for figure 7 (right-hand panel) fragments of the same original image were spliced together to remove irrelevant lanes (Supplementary Figure 2). Unwanted conditions (sulindac sulfide + insulin) were removed, since a similar condition (leptomycin + insulin) was not shown for leptomycin.

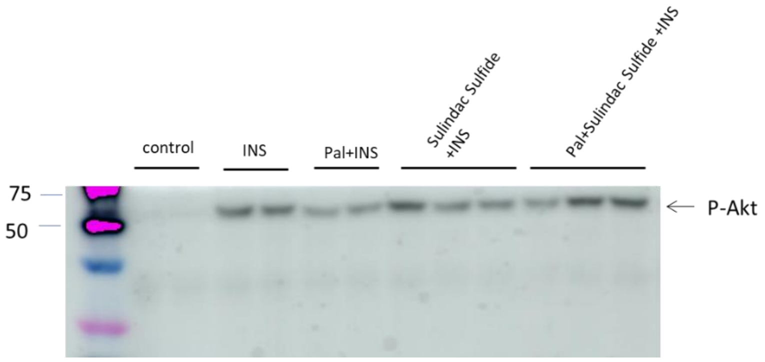

Supplement: Supplementary file 2 — Supplementary file2 (PDF 31 KB) [file 18_2024_5571_MOESM2_ESM.pdf]
